# Mindfulness and Relaxation Techniques for Stroke Survivors with Aphasia: A Feasibility and Acceptability Study

**DOI:** 10.3390/healthcare10081409

**Published:** 2022-07-28

**Authors:** Xu Wang, Lindsey Thiel, Naomi de Graff

**Affiliations:** 1 Psychology, Leeds School of Social Sciences, Leeds Beckett University, Leeds LS1 3HE, UK; 2 Speech and Language Sciences, Leeds School of Social Sciences, Leeds Beckett University, Leeds LS1 3HE, UK; l.thiel@leedsbeckett.ac.uk (L.T.); n.de-graff@leedsbeckett.ac.uk (N.d.G.)

**Keywords:** stroke, secondary prevention, post-stroke aphasia, mindfulness, relaxation, feasibility and acceptability, tailored techniques

## Abstract

Stroke survivors with aphasia (SsWA) tend to experience high levels of anxiety and stress, leading to an increased risk of recurrent strokes. Mindfulness and/or relaxation that does not require language outputs could reduce psychosocial stress; however, these approaches work best if they consist of a range of techniques and are modified to suit the needs of SsWA. Using a mixed-methods approach, we examined the feasibility and acceptability of a set of tailored mindfulness and relaxation techniques for SsWA. Nine SsWA were recruited (six men and three women, median age = 51 years). Four relaxation and mindfulness techniques which had been tailored for SsWA were filmed into a DVD/YouTube video and were given to participants together with a practice diary for home practice once daily for 5 weeks. The participants joined focus group discussions and completed a feasibility scale 5 weeks later. The participants perceived these techniques as easy, user-friendly and acceptable for SsWA in general. Although practised less often than instructed, many participants reported benefits of regular practice. The perceived relevance of these techniques to the participants’ own situations and the intention to continue varied. Future research could encourage the regular practice of self-help interventions by incorporating behavioural change techniques such as using prompts and cues.

## 1. Introduction

Around one-third of stroke survivors have aphasia, a multi-modal language disorder [1]. Aphasia can vary considerably in terms of symptoms and severity and can be classified depending on the areas of language that are impaired. For example, people with non-fluent aphasia usually have relatively spared comprehension but difficulties with spoken output, which can include short utterances, telegraphic speech and difficulties with forming sentences. People with fluent aphasia often have comprehension difficulties and fluent output with symptoms including jargon, neologisms and semantic or phonological paraphasias [2]. Aphasia can result in isolation, reduced social activity and participation as well as depression and anxiety [3,4,5]. A diminished ability to communicate is one of the most substantial stressors for people with aphasia (PWA) [6]. Anxiety was shown to be significantly higher in stroke patients with aphasia than in those without aphasia [7]. Morris, Eccles, Ryan and Kneebone (2017) recruited 111 dyads of patients with post-stroke aphasia and their caretakers who completed the Behavioural Outcomes of Anxiety scale. They found that 44% of patients had significant anxiety [8], which was higher than the 25% found in participants without aphasia [9]. Perceived stress is also greater among people with post-stroke aphasia. Hilari and colleagues (2010) reported that a higher proportion of stroke survivors with aphasia (93%) experienced psychological distress than non-aphasic stroke survivors (50%) 3 months after the stroke [10]. Compared with people with no neurological damage or with right brain damage, PWA perceived themselves as less able to reduce tension built up due to stress by relaxing or managing their thoughts [11]. 

Psychosocial stress is a primary stroke risk factor [12]. It has also been acknowledged as a risk factor for secondary strokes in a Delphi study conducted by a group of international experts in secondary stroke prevention [13]. A recent scoping review of the national stroke guidelines of 27 countries concluded that psychosocial stress is poorly addressed as a modifiable population attributable risk factor in secondary stroke prevention [14]. As noted previously, PWA and, indeed, stroke patients with aphasia report high levels of anxiety and psychosocial stress. These clinical needs warrant evidence-based rehabilitation and psychological interventions adapted for PWA [15,16]; however, survivors with post-stroke aphasia are often excluded from stroke rehabilitation studies [17], including studies addressing stroke-related anxiety or stress [9,10]. This inevitably leads to limited evidence on how best to support stroke survivors, especially those with aphasia [18], particularly for them to decrease psychosocial stress, thus reducing the risk of secondary strokes.

Difficulties in comprehension and/or spoken output in PWA may preclude the dependance of conversational-based psychological support such as ‘talking therapies’ [19]. Many practitioners do not attempt such intervention for PWA due to the uncertainty of the suitability and effectiveness of talking therapies [19]. Consequently, PWA are usually left without any form of emotional support [20]. Some research finds promising results using mindfulness and relaxation techniques for stroke participants without aphasia. These self-administered techniques could alleviate anxiety and tension after a stroke [21,22]. The benefits of mindfulness are explained through reduced stress and improved attentional control due to a decrease in rumination. Attention is shifted and re-directed to the current moment rather than focusing on past or future worries [23]. Relaxation works by generating a psychophysiological state of a decreased arousal-relaxation response, which counteracts the body’s stress response [24]. Through regular elicitation of the relaxation response, individuals become less sensitive to cortisol and epinephrine production, thus decreasing the reactivity of the sympathetic nervous system when exposed to stressful events [25]. Regular practice can help achieve and enhance the effects of mindfulness and/or relaxation [24,26].

Despite the existence of different kinds of mindfulness (e.g., yoga, meditation) and relaxation (e.g., progressive muscle relaxation, autogenic relaxation), studies usually use one single type at a time [22,27,28]. The evidence suggests that psychological interventions with multiple techniques are more effective at reducing stress than those with a single component [29]. Focusing on participants with post-stroke aphasia and their experience of mindfulness meditation, Panda et al. (2021) also proposes that a variety of mindfulness or relaxation techniques should be included when introducing such techniques to PWA to accommodate for personal preferences [30].

Yeates (2020) proposes that approaches such as mindfulness and relaxation, which focus on the body over verbal exchange, might present fewer barriers for PWA [19]. Working with 13 stroke survivors, including 5 with aphasia, a group of researchers tailored a set of commonly used mindfulness and relaxation techniques to meet stroke survivors’ needs, including those with communication difficulties. Participants in the first part of the study favoured four techniques: ‘Thinking of a nice place’, ‘Breath watch’, ‘Positive emotion’ and ‘Body relaxation’ [31]. In the second part of the study, the four preferred techniques were filmed into a DVD. With a different sample of 38 stroke survivors, Wang and colleagues [31] investigated the feasibility and acceptability of these four techniques. After a period of practice, 18 survivors, 2 of whom had communication difficulties, participated in focus group discussions/interviews. The participants found these techniques acceptable, user-friendly and beneficial (e.g., relaxing). They found that a once-a-day practice frequency could make practising more feasible and preferred having choices of multiple techniques rather than a single technique.

As participants need to follow verbal instructions alongside imitating the actions of the demonstrator/instructor, it may be expected that participants with severe aphasia and an impairment in auditory comprehension may have some difficulties carrying out mindfulness tasks [32]. Spoken language difficulties may be less restrictive, as participants are not usually expected to produce any language in this type of tasks, apart from needing to ask questions when they do not understand the content [19]. However, a small body of existing research has indicated that participants with mild, moderate and severe language impairments can learn and benefit from mindfulness training and activities. For example, Orenstein et al. (2012) measured the effects of mindfulness meditation on divided attention and language in three participants with mild-to-moderate post-stroke aphasia [32]. Despite no improvements being found for these measures, the participants were able to learn and complete the meditation without any difficulty and reported that they felt more ‘relaxed’ and ‘peaceful’ post training. It was also highlighted that PWA with mild-to-moderate comprehension deficits can learn mindfulness. In a single case study, Laures-Gore et al. (2016) trained a participant with moderate post-stroke aphasia in mindfulness meditation, which led to changes in psychophysiological measures, including heart rate and heart rate variability, as well as linguistic and cognitive measures such as word productivity and increased attention [33]. Dickinson et al. (2016) provided a mindfulness programme to a stroke survivor with severe non-fluent expressive aphasia and largely intact comprehension with the aim of reducing anxiety [34]. They found reduced scores on the Beck Anxiety Inventory and increased scores on the naming, picture description and repetition assessments following this programme. Marshall et al. (2017) compared mindfulness meditation training for a group of five stroke survivors with a range of types and severities of aphasia to a waitlist control group who completed a mind-wandering activity, also in a group [35]. This study confirmed that PWA can be trained in mindfulness meditation, although no statistically significant changes were found in terms of physiological measures (salivary cortisol, heart rate and heart rate variability). Panda and colleagues (2021) interviewed five people with mild-to-moderate post-stroke aphasia on their past experience of attending mindfulness meditation groups [30]. They found that mindfulness meditation can be learned by PWA, although it is a ‘gradual’ journey. Participants also reported that focusing on activities that do not require a language output or being physically active, such as mindfulness, allowed them to move their attention away from their impairment.

Other studies have investigated the effects of relaxation on reducing anxiety for PWA. Murray and Ray (2001) used progressive muscle relaxation (PMR) and guided imagery (GI) as well as syntax stimulation with a person with moderate non-fluent aphasia [36]. The participant demonstrated improvements in his spoken language, particularly when the relaxation training preceded the syntax stimulation. Reviews by Murray and Kim (2004) [37] and Laures-Gore and Marshall (2004) [38] have found some evidence for complementary and alternative treatments such as relaxation and acupuncture. The benefits have included improvements in linguistic, cognitive and emotional symptoms when combined with behavioural treatments, although both reviews concluded that there are significant methodological weaknesses and inconsistencies in the findings within this body of research.

More research is needed into the support of anxiety and psychosocial stress in PWA [39]. This is particularly important during and after the COVID-19 pandemic, as PWA were found to experience a significant increase in anxiety symptoms compared with the pre-COVID period [40]. Most studies of psychological interventions are conversational-based and are not suitable for PWA. Among the few non-talk-based psychological interventions, behavioural therapy, which is accessible for PWA, can reduce depression but not anxiety and/or stress [41]. Mindfulness and relaxation techniques are potentially beneficial to decrease anxiety and/or stress and do not require a language output or being physical active, which is positive for PWA [30]; however they are often poorly optimised for stroke survivors, especially those with communication difficulties [42]. None of the previous studies focusing on PWA tailored the mindfulness or relaxation to suit the needs of PWA, nor did they incorporate multiple techniques to cater to different approaches. Moreover, there is a distinct lack of research to date on the feasibility of using relaxation or mindfulness techniques to reduce anxiety/or stress for people with post-stroke aphasia [31], even though they are more likely to suffer from anxiety and stress than those without aphasia.

To address the knowledge gap and the unmet needs of the largely ignored group of PWA, this study examined the feasibility and acceptability of a set of tailored techniques to reduce stress and anxiety for PWA. This study aimed to establish the following: (1) the acceptability of mindfulness and relaxation techniques for participants with post-stroke aphasia; (2) the feasibility of using these techniques via a DVD/YouTube link; (3) the feasibility of daily practice within the study period; and (4) any modifications to the techniques following the participants’ individual needs.

## 2. Methods

### 2.1. Design

This study adopted a mixed-methods design using both qualitative and quantitative approaches. It was deemed appropriate for answering the research questions about the feasibility and acceptability of using these techniques for PWA. We used questionnaires, participants’ practice diaries and focus group discussions to collect the data.

### 2.2. Intervention

Four mindfulness and relaxation techniques were used in this study: (1) positive emotion; (2) body relaxation; (3) thinking of a nice place; and (4) breath watch. They were favoured by participants in a previous study and were tailored to suit stroke survivors’ needs, including the needs of those with communication difficulties [31]. Details on tailoring the techniques for PWA can be found in our previous paper. The techniques were filmed into a 15 min-long DVD starting with a brief introduction on the benefits of mindfulness and relaxation to promote participants’ engagement [43]. The DVD content was also uploaded onto YouTube so participants could choose to watch the YouTube video online if they did not have a DVD player. This combined delivery mode was suggested by participants in our previous study [31]. The descriptions of these techniques are listed in Table 1.

We also provided participants with aphasia-friendly instructions on using the techniques at home. As suggested by Richardson and colleagues (2008), self-help materials should be user friendly and suit users’ needs [45]. The instruction advises participants on how they could prepare before practising (i.e., allow themselves 15–20 min in a quiet place free from disturbances), what they need to do and, finally, how they could end the session (i.e., outlining that at the end they would hear a beep).

### 2.3. Measures and Apparati

#### 2.3.1. Feasibility and Acceptability Measure

A 5-item feasibility scale was designed to measure participants’ perception of the feasibility and acceptability of practising these techniques at home. Participants chose from 1 (very difficult/not at all feasible/not at all relevant) to 5 (very easy/very feasible/very relevant). A higher score represents a more favourable outcome.

A self-report practice diary was used to measure participants’ engagement of the techniques at home. The tick-box diary had two options for each day: ‘no practice’ or ‘practised’.

#### 2.3.2. Background Measures

To gain an overview of the participants’ language and cognitive skills, the Frenchay Aphasia Screening Test (FAST) [46]; the Western Aphasia Battery (WAB-R) [47]; and subtests of the Cognitive Linguistic Quick Test-Plus (CLQT+) [48] were used. The FAST is designed to screen comprehension, expression, reading and writing and to determine the presence of aphasia. A FAST score of less than 27 for people below the age of 60 indicates the presence of aphasia.

The CLQT+ provides information about the cognitive-linguistic functioning of people with neurological disorders. Three sub-tests were chosen to assess participants’ language and memory, as these skills were predicted to be important for taking part in the intervention. In the ‘Personal Facts’, participants were asked to recall personal information about themselves. This involves understanding the question (language comprehension), remembering the information and being able to verbally express the response (expressive language). The ‘Story re-telling’ sub-test assesses attention, working memory, language comprehension and verbal production. The participant listens to a story, retells the story and answers yes/no questions about the story. Finally, the ‘Generative Naming’ subtest assesses skills in working memory and expressive language (word retrieval). This involves participants saying as many words as they can beginning with ‘m’ and then naming as many animals as they can. A higher score in the subset indicates a higher level of skills in that specific area. Each subset has a cut-off score to indicate the presence (below the cut-off score) or absence (above the cut-off score) of the problem in that area (see Table 2).

The Western Aphasia Battery Revised (WAB-R) is an assessment of the linguistic skills and non-linguistic cognitive skills of adults with aphasia. The scores reported for this study were those for spontaneous speech (conversation and picture description), auditory verbal comprehension (yes/no questions, auditory word recognition and sequential commands), word repetition, naming and word-finding (object naming, word fluency, sentence completion and responsive speech), apraxia (imitating actions), constructional, visuospatial and calculation (drawing, block design, calculation and Raven’s Coloured Progressive Matrices) [49], as well as the Aphasia Quotient (AQ), which indicates the severity of oral language [50]. AQ combines scores on spontaneous speech, auditory verbal comprehension, repetition and naming and word finding. These data provide a profile of participants’ expressive and receptive language skills, as well as an indication of their cognitive abilities.

Participants’ functional ability and independence was measured by the 10-item Barthel Activities of Daily Living Index [51]. The scale provides a total functional ability score, which ranges from 0 (less severe disability) to 20 (more severe disability). The total score can also be categorised into five categories indicating different levels of functional ability [52]: very severely disabled (0–4); severely disabled (5–9); moderately disabled (10–14); mildly disabled (15–19); and independent (20).

### 2.4. Recruitment

Nine participants were recruited via the Service Users and Carer group at the authors’ university and local stroke support groups in West Yorkshire, UK. To be included in this study, participants had to: (1) have a diagnosis of a stroke and present symptoms of aphasia; (2) have since been discharged from the hospital to live in the community; (3) be over 18 years old and English literate; and (4) not have severely impaired comprehension, due to the need to be able to understand the purpose of the study and to follow instructions. People were excluded if they felt that they had a medical, physical or cognitive condition that may affect their capacity to consent or participate in the study.

### 2.5. Participant Characteristics

The participants were nine stroke survivors with aphasia (six men, three women) with a median age of 51 years (range: 40–60 years). Six participants self-identified as White British, one as Chinese, one as mixed ethnicity and one as Black African. All participants had just one stroke. The time between their stroke and the commencement of the project ranged between 1.5 to 13.5 years, with a median length of 4 years. Most participants were independent or mildly disabled, although one survivor was moderately disabled. Most of the participants had high scores on the FAST, and some were at the ceiling; however, it is important to note that all of the participants described themselves as having aphasia and reported that aphasia impacted their quality of life, wellbeing and level of activity and participation in everyday life. In terms of the CLQT+ Personal Facts, all participants were at the ceiling, apart from DB, who has more severe language difficulties. Over half of the participants showed sufficient memory, auditory comprehension and expressive language to obtain a score of the cut-off or above for the Story Retelling subset, whilst those that scored below the cut-off had difficulties with language or memory. Relating to Generative Naming, only four participants scored at or above the cut-off, which seems to reflect the expressive language difficulties of the other participants. The participant DB showed difficulties across language assessments. Table 2 shows the participants’ demographic information and scores on the Barthel, FAST and the three subsets of the CLQT+.

With regard to the scores of WAB-R, we have missing data for DB and GD, so we are only reporting the WAB-R scores for seven participants. As can be seen in Table 3, the participants varied in their scores for all subtests. In terms of the Aphasia Quotient (AQ), one participant (JP) scored above the cut-off (93.8). Most participants could be described as mild (a score of 76 or above), and one participant’s (IA) AQ of 73.8 indicates moderate aphasia (51–75). KD, JP and JR all had high scores in tasks that involved spoken output, while others showed more impaired spoken language skills in either naming and word-finding or spontaneous speech. In terms of auditory verbal comprehension, those with the lowest scores were KN (180/200), PB (179/200) and IA (160/200). On the apraxia sub-test, the participants scored 50–60, indicating minimal difficulties in imitating actions. On the constructional, visuospatial, and calculation subtests, which assess non-linguistic cognitive skills, the participants’ scores ranged between 81 and 96 /100. In summary, the WAB-R showed that the seven participants had mild-to-moderate aphasia and no indication of severe cognitive impairment.

### 2.6. Procedures

Ethics approval was granted by the authors’ institutional research ethics committee. All participants gave written consent before taking part. Participants were invited to meet as a small group in a meeting room at the university. They met at two time points: T1 and T2 (5 weeks after T1).

The participants completed the questionnaires individually. Assistance on how to complete these measures was provided when required. A speech language therapist (R.O.) who was not part of the research team and L.T. assessed the participants’ language and cognitive skills using the FAST, the CLQT+ and the WAB-R. The participants watched the DVD and practised all four techniques, following the instructions in their small group setting. All the participants had sufficient comprehension to follow the DVD and confirmed that they could follow the instructions. They then took the DVD home (or a link of the YouTube video) and were instructed to practise the techniques once a day for five weeks and to record their practice in a diary provided. This practice frequency was suggested by participants from our previous study 27. About 2–3 weeks after T1, XW contacted participants to answer any queries regarding their practice and to remind them of the date of T2. None of the participants sought additional help regarding the techniques during the study.

The participants attended a second meeting five weeks later (T2) in a meeting room at the university. They watched and practised all four techniques together for a second time. After a short break, focus group discussions were conducted (around 45 min long each), and the feasibility scale was given to participants to fill in.

The focus groups explored the participants’ views on the acceptability and feasibility of practising these techniques at home. Eight PWA participated in the group discussions (KN did not attend the T2 meeting). Four caretakers were also present during the discussions. Focus groups 1 and 2 were run by NDG and XW, respectively, using a focus group topic guide. All discussions were audio-recorded with the participants’ consent. The participants were asked about the following: 1. their experiences with practising the techniques at home (e.g., could they practise them?); 2. what motivated or demotivated them; 3. whether they observed any changes to stress and anxiety while using the techniques; and 4. any recommendations for the future regarding using the techniques.

### 2.7. Data Analysis

One participant did not attend the T2 meeting; this left us with the data of 8 PWA for the feasibility scale, practice diary and focus group data. Feasibility was considered in terms of adherence rate using the practice diary and the feasibility scale [53], which were analysed mainly descriptively. The audio-recordings of the focus group data were transcribed verbatim. We conducted a thematic analysis using the steps defined by Braun and Clarke [54] to report key themes on the perceptions of the acceptability and feasibility of practising these techniques. The datasets were coded by giving every meaningful piece of text a descriptive label indicating the meaning of that piece of text. The next step was to group those pieces of text which had been coded similarly and apply a theme name to those pieces of text to capture a pattern. At the end of this process, we arrived at five themes concerning the participants’ experiences of practising these techniques and their views on the acceptability and feasibility of these techniques delivered on a DVD.

## 3. Results

### 3.1. The Practice Diary Data

Two participants noted that they went on holiday for a week and thus could not practise the techniques during holidays. In each case, an average practice frequency of that person over the rest of the time between T1 and T2 was used for the holiday period. The median practice frequency was 20 times over 5 weeks (range = 11–28; interquartile range = 17–26), which indicated that the participants practised about four times a week over 5 weeks. The total number of practice sessions did not correlate with the participants’ age, time since stroke, Barthel scores or any of the WAB-R subsets scores. However, it negatively correlated with the FAST total score (rs = −0.75, *p* = 0.03) and the CLQT Generative Naming scores (rs = −0.82, *p* = 0.01). Participants with less severe aphasia, a better working memory and expressive language skills tended to practise less, often comparing themselves to their peers with more severe aphasia and poorer Generative Naming skills.

### 3.2. The Feasibility Scale Data

Turning to the feasibility scale, the median and the interquartile range (IQR) scores for intervention feasibility are presented in Table 4. The participants reported that it was very easy to use these techniques as well as the DVD/YouTube link. They reported that daily practice was fairly feasible, although remained neutral on their intention to keep using these techniques after the study. However, they found that these techniques were not completely relevant to their own situations.

### 3.3. Qualitative Focus Group Data

Five themes were generated concerning the acceptability and feasibility of these mindfulness and relaxation techniques delivered on video. They were: (1) the techniques are easy to use and suitable for PWA; (2) barriers for daily practice; (3); varied feelings about the effect of practising these techniques; (4) the intention to continue with the techniques depends on the perceptions of these techniques, personal hobbies and current life status; and (5) further modifications to make the techniques better. These themes and selected quotes are presented in Table 5.

A general pattern reported by the survivors was that the techniques were easy to practise. They also found the DVD or the YouTube link easy to use. Many survivors reported that these techniques are suitable for PWA. One participant (PB) even practised these techniques in a supermarket when she felt anxious. This theme was substantiated by the high median score of the first and third question on the feasibility scale (5—very easy to practise/use) and by the fact that no participant sought additional help or raised concerns during the telephone contact between T1 and T2.

Although the participants reported that these techniques are easy to use, a few of them also acknowledged some barriers for the daily practice of these techniques. Busyness (IA and EH, who had their stroke 13 and 11 years ago, respectively), fatigue (EH) and physical pain (DB) made practising daily difficult. DB’s sister also added that DB ‘had a lot of other things going on’, as he had not yet fully settled in the UK. This was the reason that DB had difficulty practising these techniques every day. Other participants talked about their experience of building a routine (e.g., before bedtime) so that the daily practice became easier.

Participants made mixed comments regarding how they felt about the effect of practising these techniques. Many survivors or their caretakers present at the discussion observed that they (the survivors) were more relaxed after practising. However, two survivors did not agree. GD did not like these techniques and even reported feeling ‘stressed out’ about the thought of doing them. JP commented that these techniques are boring and thus did not continue to practise them.

The participants’ perceptions of these techniques, personal hobbies and current life status seem to be associated with their intention to continue practising them after the study. Many participants liked at least one of the techniques and/or felt calmer after practising them; thus, they wanted to continue practising them. For example, KN liked these techniques as they gave him ‘a good sleep in the night’; thus, he would keep using them after the study. On the contrary, participants who did not like the techniques reported that they would stop practising. GD commented that he found it difficult to concentrate while practising, particularly for ‘Breath Watch’—‘I can’t stop thinking. That’s my head, I guess…’ Training to be a security guard, GD also stated that he would rather carry on with hobbies such as ‘Wing Chung’ than continue with relaxation and mindfulness. Similarly, JP thought these techniques are boring, and he liked cycling on his own for relaxation. JP would not continue practising. IA, who had a stroke 13.5 years ago, commented that they ‘don’t need it (these techniques)’ for their life at the moment.

The participants also suggested possible ways to make these techniques better. The survivors appreciated that they could choose different technique(s) to practise; however, it would be better to have four different DVDs so they can just practise the technique they really liked at a time. Some survivors suggested changing some of the words. For example, ‘your shoulder is feeling heavy’ is better worded as ‘your shoulder is feeling relaxed’, as they associated ‘heavy’ with gravity. Some survivors would like a sound ‘signal’ as a cue at the end of the DVD so they would know that it had finished.

## 4. Discussion

### 4.1. Feasibility

Feasibility was considered in terms of the easiness of delivery and practice, the adherence rate and the scores from the feasibility scale. No concerns were raised, and no participant sought additional help during the study. The participants reported that these techniques were easy to use, and the DVD/YouTube video was simple and straightforward to use at home. Similarly, using the feasibility scale, the participants scored ‘very easy’ for questions on the easiness of practising techniques and the easiness of using the DVD/YouTube video.

The data from the feasibility scale showed a median score of ‘feasible’ for practising these techniques every day. However, the results from the practice diary revealed that techniques were practised less often than the required daily frequency. On average, the participants practised four times a week over five weeks. The total number of practice sessions was not associated with participants’ abilities in auditory comprehension or imitating actions; however, the participants tended to practise less if they scored higher on memory (the CLQT+ Generative Naming scores) and language abilities—in particular, expressive language (FAST total score and the CLQT+ Generative Naming). It is worth noting that the severity of language impairment was the most significant factor impacting PWA spending time out of the house and engaging in social and community activities [55]. It is possible that participants with a better working memory, expressive language and, indeed, less severe aphasia were engaging with more activities outside their house. Thus, finding time to practise these techniques at home became more challenging for them. Indeed, busyness was one of the main reasons preventing daily practice that was reported by the participants. Likewise, busy lifestyles and a lack of time were reported barriers for engaging in relaxation and/or mindfulness techniques among stroke survivors [31] and patients with cancer [56]. Fatigue, physical pain and a lack of personal interest in mindfulness and relaxation were also reported reasons for not practising every day in this study. Similar factors were suggested as barriers to practising relaxation and/or mindfulness in other studies [56,57]. Some participants in the present study found that building a daily routine helped them with everyday practice. This echoes the findings from a study where a group of young people used a mindfulness app [58]. One of the enabling factors for those young participants to engage in mindfulness was developing daily routines.

### 4.2. Acceptability

One element of acceptability comes from the participants’ comments providing preliminary evidence of the usefulness of these techniques [59]. Despite practising less than daily, most participants and their caretakers reported themselves or observed the PWA as being more relaxed and calmer and as sleeping better after practising. For example, one participant used these techniques to help keep calm in a noisy and potentially stressful environment. Similarly, Orenstein et al. [32] reported that their participants with aphasia felt more ‘relaxed’ and ‘peaceful’ after practising mindfulness. Two participants in our study felt the techniques were boring or ‘not for them’ and thus did not find practising them beneficial.

The participants commented positively on the variety of techniques included and the fact that they could choose to practise the one(s) they preferred. They stated that each stroke is different and that different techniques suit different people best. When assessing 149 participants’ engagement in a web-based mindfulness intervention designed for 9-1-1 telecommunicators, Kerr et al. (2019) found many of their participants appreciated the variety in the intervention content, allowing them to choose what worked best for them [60]. Previous research also suggested that, in comparison with single-component techniques, those with multiple components are better suited for patients and are more beneficial [29,30,61].

Another element of acceptability is the perceived suitability of these techniques and the intention to continue afterwards [62]. Most participants in the focus groups perceived these techniques as suitable for aphasic stroke survivors in general. Nevertheless, the feasibility scale data showed that these techniques were not completely relevant to the participants’ own situation. They were also somewhat neutral regarding their intention to continue with these techniques after the study. These results could be because some participants did not like relaxation and/or mindfulness. Some reported that these techniques were not necessary in their life at the moment because they had a stroke over 13 years ago. The evidence suggests that psychological distress for PWA seems to decline with time after the stroke [63]. This could explain why this participant felt that they did not need these techniques now. However, the degree of the perceived relevance of these techniques did not correlate with how long ago participants had their stroke (rs = −0.23, *p* > 0.05), nor did the intention to continue or the time since the stroke (rs = −0.40, *p* > 0.05). The perceived relevance and the intention to continue were significantly associated with the CLQT+ General Naming subtests scores (rs = −0.72, *p* = 0.046; rs = −0.88, *p* = 0.004). This indicates that PWA with better working memory and expressive language deemed these techniques less relevant and felt less likely to continue using them. Evidence shows that PWA want to engage with others/groups/the community [15]. Those with a better communicative ability tended to spend more time engaging in social activities [55], thus finding these individually practised techniques less relevant. This raises an interesting question: is practising these techniques in a group setting more relevant for PWA who have a less severe language impairment? Panda et al. (2021) [30] argued that medication interventions for PWA may be more helpful if delivered in a group environment rather than individually. However, all their participants had attended meditation groups regularly. Perhaps it is not surprising that they favoured a group setting.

Although the median score was ‘neutral’ on the item of intending to continue with these techniques, it was encouraging to see that many participants in the focus groups would like to continue. They liked at least one of the techniques and/or found practising them useful. Nevertheless, three PWA stated clearly that they had stopped practising them during the study and/or did not intend to continue afterwards. A lack of interest, a preference for other activities such as physical activities and negative perceptions of mindfulness were reported reasons for not practising and continuing. In a similar vein, previous studies also found that negative attitudes towards mindfulness and a lack of interest are barriers for engaging with relaxation and/or mindfulness practice [57,58].

The participants also provided valuable feedback on how to improve the presentation of these techniques. This includes changing some of the wording, making four separate videos for each technique and having a ‘signal’ sound at the end of the video.

### 4.3. Limitations

Although all of our participants described themselves as having aphasia and reported that their life had been affected by aphasia, most of them scored high on the FAST. It is unclear how our results might apply to stroke survivors with more severe aphasia. Future studies should aim to select participants with a range of types and severities of aphasia.

It is encouraging to see that the participants in this study came from different ethnic backgrounds; however, the volunteers were largely self-selected and recruited from local stroke support groups and the Service Users and Carer group at one university. Although varying in terms of the length of time since their stroke, the participants, on average, had their stroke several years ago. This might be the reason for the moderate-to-high level of daily living and the less severe level of aphasia. This group of participants may differ from those aphasic stroke survivors who did not volunteer to participate. Manning et al. (2019) suggested that people living successfully with aphasia are more likely to volunteer in activities [15]. It is unknown how our findings might apply to those who are less motivated to take part in research and those who have been recently discharged from hospitals.

## 5. Conclusions

We addressed a research gap and a clinical need by exploring the feasibility and acceptability of a psychological intervention for PWA, who are often excluded from stroke rehabilitation studies and studies addressing post-stroke anxiety [17]. These mindfulness and relaxation techniques were tailored to suit stroke survivors’ needs, including the needs of those with aphasia. It is promising to see that the participants, who had mild-moderate aphasia, perceived the techniques as feasible and acceptable for people with post-stroke aphasia in general. Future studies could highlight the importance of a routine and help participants set up a daily routine to facilitate regular practice. A systematic review of stroke behavioural interventions suggested a lack of a theoretical underpinning for the development of self-help interventions [64]. Future research could also apply behavioural change theory and techniques in the form of prompts and cues to encourage the regular practice of a self-help intervention [65]. Furthermore, future studies could recruit participants with more severe aphasia to determine whether they would also find the techniques to be feasible and acceptable.

Although practised less often than instructed, many participants still reported benefits after practising. Nonetheless, the perceived relevance of these techniques to participants’ own situations and the intention to continue varied and depended on the attitudes towards the techniques, personal hobbies and current life status. Our findings provide a good foundation for future studies to further improve mindfulness and relaxation, which are meaningful for stroke survivors with aphasia in order to reduce anxiety and stress. Ultimately, lowered psychosocial stress will decrease the risk for recurrent strokes.

## Figures and Tables

**Table 1:** Description of the mindfulness and relaxation techniques used in this study.

Name of the Technique	Description
Positive emotions (relaxation-guided imagery)	Asks participants to generate a positive emotional experience by imaging a ball of light filling them with rays of happiness and love.
Body relaxation (autogenic relaxation)	Focuses on different parts of the body and concentrates on relaxing that part. Participants do not need to physically move any body parts.
Thinking of a nice place (relaxation-guided imagery)	Asks participants to imagine a place where they were happy in the past. This technique, along with positive emotions, incorporated principles from positive psychology which involve mental exercises that cultivate positive mood states [44]
Breath watch (mindfulness)	Focuses on breathing and noticing their breath as they breathe in and out. They were asked to not change their breathing but rather to just watch it happen.

**Table 2:** Participants’ demographic information and scores on Barthel, FAST and three subtests of CLQT (*n* = 9).

Participants	IA	DB	PB	GD	EH	KM	KN	JP	JR	Maximum Total Score	Cut- Off
Age	59	50	40	50	60	54	49	51	55	-	-
Gender	M	M	F	M	F	F	M	M	M	-	-
Time since stroke (years)	13	1	9	3	11	4	2	3	4	-	-
Occupation before stroke	Electrician	Sales representative	Customer service representative	Trainee security guard	Secretary	Teacher	Senior lecturer	Communication analyst	IT consultant	-	-
Barthel total	20	18	15	20	13	17	18	20	20	20	-
FAST total *	25	20	27	29	29	26	24	30	29	30	27 *
CLQT + Personal Facts	8	7	8	8	8	8	8	8	8	8	8
CLQT + Story Retelling	5	4	3	6	4	7	6	7	8	10	6
CLQT + Generative Naming	5	2	4	7	4	3	2	5	3	9	5

* Note: A FAST score less than 27 for people below the age of 60 indicates the presence of Aphasia.

**Table 3:** Participants’ scores from subtests of WAB-R (*n* = 7).

Participants	IA	PB	EH	KM	KN	JP	JR	Maximum Score
Spontaneous speech	13	14	18	19	15	19	19	20
Auditory verbal comprehension	160	179	192	191	180	198	200	200
Repetition	76	77	72	94	86	96	89	100
Naming and word-finding	83	93	70	88	65	98	88	100
Aphasia quotient	73.8	80	83.6	93.6	78.2	96.6	93.4	100
Apraxia	57	50	59	60	56	58	56	60
Constructional, visuospatial and calculation	81	81	96	95	95	93	94	100

**Table 4:** Median and IQR scores for intervention feasibility (*n* = 8).

Question	Median and IQR	Score Values
1. How easy was it to practise these techniques?	5 (4–5)	1 = very difficult, 5 = very easy
2. How feasible was it to practise these techniques every day during the study?	4 (2.5–5)	1 = not at all feasible, 5= very feasible
3. How easy did you find using the DVD/YouTube?	5 (4.25–5)	1 = very difficult, 5 = very easy
4. How relevant did you think the techniques were to your situation?	2.5 (1.25–4.75)	1 = not at all, 5 = very relevant
5. Do you intend to continue to practise these techniques in your daily life after the study?	3.5 (1–5)	1 = not at all, 5 = very much

**Table 5:** Selected quotes and overall descriptive themes from the focus group data.

Themes	Sample Quotes
The techniques are easy to use and suitable for PWA	Morrison’s, at 2 o’clock in the afternoon, kids. Screaming kids. And it’s all right, because mindfulness and I’m doing it every day, to take my mind off things, and problems. (PB)
	**And were you able to use the techniques, in Morrison’s? (Interviewer)**
	Yes, yes. (PB) **…And was it easy enough to use a DVD, or a YouTube link? (Interviewer)** Yes (EH, IA, GD, JP, JR, PB, DB). **…So, it seems everybody found it quite easy to use. And do you think the techniques were suitable for people with aphasia, then? (Interviewer)** PB: Yes. EH: Oh, yes. Yes. JR: Yes.
Barriers for practising daily	**Were there any things that made you, sort of—made it hard for you to practise every day? You know, it can be difficult to fit things in. (interviewer)** Yes. I was tired, and I was so busy. Yes. (EH) But I think that’s just because it’s been all so unsettled, because we only came here in February, and then decided to stay. There are so many logistics, that… You know, we’re not settled yet. (DB’s sister) **No, you’ve got a lot…(interviewer)** And then the sciatica, which…(DB’s sister)
Varied feelings about the effect of practising	Mum, what do you think? Has it (practising the techniques) changed me? (PB) You are calmer. (PB’s mum) …Yes. I feel relaxed, but calmer, yes (PB)
	Well, it (practising the techniques) gives you a good sleep in the night. It was nice, so I kept doing it. (KN)
	Or the sun in my brain, it (positive emotion) was very useful for me. (EH)
	Yes. I was going—I was stressing out, about… I was stressing out at the thought of having to… (GD) **To do it? (interviewer)** Yes. (GD) **So the idea of having to sit down, and go through it, you just thought {groans}. (interviewer)** The thought of it, I’m stressed. {Laughter}. (GD)
The intention to continue with the techniques depends on the perceptions of these techniques, personal hobbies and current life status	In my head, I’ve got the techniques here. So, you know, if I have to use them, I will. But…(GD) **Would you use them? (interviewer)** No. (GD) **No? Why not? (interviewer)** Work out, you know? Work out, yes. (GD) **So you’d rather do something physical? (interviewer)** Yes, yes. (GD)
	No. It was fine the first five days. And then, I decided it was boring… (JP) **Does anyone think they will keep going with it? (interviewer)** I think I will (KN). I had a stroke with different, long time ago, one year, two years intense, intense but me especially, there’s me that’s all. I don’t need it (using these techniques)... (IA)
Further modifications to make the techniques better	It depends on the person. And I think it would be nice to have four different DVDs (JR) Yes. (PB) Yes? To choose from. Rather than doing them all… (JR) But the language on the video is—it’s not good, you know? (GD) **In what way is it not good? (interviewer)** Heavy. (GD) **What do you mean? Can you describe what you mean by ‘heavy’? (interviewer)** Your hand, okay? It’s heavy…That’s the… No, it doesn’t work. (GD) **Okay. Is there a word that you would use instead, just out of interest? So I’m just trying to work out…(interviewer)** Well, like, relaxing. (GD) **Relaxing? Yes. …(interviewer)** But heavy? No. That’s gravity. (GD) Yes, good idea. It’s good, that. (IA)

## Data Availability

The data presented in this study are available on request from the corresponding author. The data are not publicly available due to the participants’ privacy.
